# Cumulative Effects of Low-Level Lead Exposure and Chronic Physiological Stress on Hepatic Dysfunction—A Preliminary Study

**DOI:** 10.3390/medsci8030030

**Published:** 2020-08-05

**Authors:** Emmanuel Obeng-Gyasi

**Affiliations:** Department of Built Environment, North Carolina Agricultural and Technical State University, Greensboro, NC 27411, USA; eobenggyasi@ncat.edu; Tel.: +1-336-285-3132

**Keywords:** allostatic load, liver lead, lead-exposure, chronic stress, psychosocial stress

## Abstract

Chronic physiological stress and hepatic injury were explored in this cross-sectional study using data from the National Health and Nutrition Examination Survey (NHANES) 2007–2010. Lead exposure was measured using Blood Lead Levels (BLL), which were divided into quartiles of exposure based on the distribution within the database. Allostatic load (AL), a variable representing chronic physiological stress, was operationalized using ten clinical markers. The geometric mean values for markers of liver injury of interest (a) Aspartate Aminotransferase (AST), (b) Alanine Aminotransferase (ALT), (c) Alkaline Phosphatase (ALP), and (d) Gamma glutamyl-transferase (GGT) were explored in quartiles of lead exposure. Associations between AL and AST, ALT, ALP, and GGT among those exposed to lead were analyzed using linear regression models. In examining lead exposure in increasing quartiles, the geometric mean of the liver injury markers showed significant elevations as lead exposure levels increased. Simple linear regression revealed AL was positively associated with several markers of hepatic injury in all degrees of lead exposure. This study demonstrates the potential dangers of social and environmental exposures to liver health.

## 1. Introduction

Lead exposure may come about due to exposure from lead-contaminated paint, water, jewelry, candy, soil, and dust [1,2]. The accumulation of lead in the body begins in the womb [3], builds up in the bone over time, and induces pathology in numerous organ systems within the body [4,5,6,7,8,9]. This process can alter the life course of exposed individuals by downwardly altering their education outcomes, income, and behavior [10,11].

No level of lead exposure is safe, and even very low levels of exposure may significantly harm the health of individuals [12]. Little is known about the cumulative effects of low levels of lead exposure on the liver and even less about the cumulative role of lead exposure and the exposure to chronic physiological stress on the liver.

The liver is the largest organ in the human body and has three vital functions (a) detoxification—where it recovers and eliminates many toxins and toxicants, (b) synthesis—where it metabolizes critical macromolecules such as carbohydrates, fats, and proteins, and produces bile and critical coagulation factors, and (c) storage—where the liver stores essential vitamins such as (A, D, E, and K) and glycogen, which is critical for the energy needs of the body.

Studies examining the hepatotoxicity of lead have found that lead exposure modifies cholesterol and xenobiotic metabolism, and can be involved in pathological mechanisms bringing about hepatic hyperplasia [13].

Liver injury can be evaluated by examining clinical markers of liver damage such as Alanine aminotransferase (ALT), Aspartate aminotransferase (AST), Alkaline phosphatase (ALP), and Gamma-glutamyl transferase (GGT). Serum activity of these markers is often a reflection of the physiological state of the liver, and their activity in the blood often indicates the severity of cellular damage [14,15]. Lead exposure has been shown to alter these makers [16,17].

Stress can result from a mixture of biological and behavioral reactions [18] that result in activation of the hypothalamic–pituitary–adrenocortical (HPA) axis and stimulation of the sympathetic nervous system (SNS) with increased levels of epinephrine, cortisol, and pro-inflammatory cytokines [19], all of which could induce liver dysfunctions [20]. Additionally, stress helps to promote unhealthy behaviors such as alcohol intake, smoking, and poor diet, which are also associated with adverse liver pathologies.

Allostatic load (AL) can serve as a marker of chronic systemic physiological stress in that it collects markers from various physiological systems within multiple organ systems; this helps to understand the biological burden that stress has on the human body [21,22,23,24].

This study seeks to explore the role of chronic physiological stress and low-level lead exposure on hepatic function in humans.

## 2. Materials and Methods

### 2.1. Hypothesis

This study hypothesizes that increasing levels of low-level lead exposure and chronic physiological stress promote liver injury. The objective of this study was therefore to examine the effects of AL on AST, ALT, ALP, and GGT in adults exposed to various degrees of lead.

### 2.2. Research Design

This cross-sectional study was based on analysis of data from the National Health and Nutrition Examination Survey (NHANES). The relationship between AL, and markers of liver injury ALT, AST, ALP, and GGT among individuals exposed to differential lead levels as defined by blood lead levels (BLL) were explored using NHANES 2007–2010. NHANES data is a stratified, multistage probability sample of the civilian non-institutionalized individuals within the United States.

#### 2.2.1. Operationalizing Allostatic Load

Based on prior studies [25,26], AL was operationalized via a cumulative index of dysfunction across multiple physiological systems such as the cardiovascular system (Systolic Blood Pressure—SBP, Diastolic Blood pressure—DBP, triglycerides, High Density Lipoprotein—HDL cholesterol, total cholesterol); the inflammatory system (C-reactive Protein—CRP for systemic inflammation); and finally the metabolic system (Albumin, Body Mass Index—BMI, hemoglobin A1C, and Creatinine Clearance). These markers were divided into quartiles based on their distribution within the database with high-risk for each biomarker considered the top quarter in the distribution for all markers apart from the markers for albumin, creatinine clearance, and for HDL cholesterol, for which the lowest quarter of the distribution was the highest risk [8,27,28,29,30,31,32]. Each participant within the data was assigned a value of 0 if in the lower-risk category with a value of 1 for those in the high-risk category to calculate a total AL value out of 10 by summing all ten variables of interest. The additive model of allostatic load used in this study predicts mortality and morbidity outcomes more accurately than individual components of AL do and comparably with similar aggregate measures of multiple physiological-system activity [33].

#### 2.2.2. NHANES Data Collection Procedures

Demographic information was collected during an in-home interview using a standardized questionnaire with clinical and anthropometric data collected at the mobile examination center (MEC). Blood was drawn from participants antecubital vein with Inductively Coupled Plasma Mass Spectrometry (ICP–MS) used to analyze it for BLL. Urine was analyzed for urinary creatinine and albumin at the University of Minnesota. A solid-phase fluorescent immunoassay was used to measure Urine albumin was measured using a Sequoia-Turner Digital fluorometer (Sequoia-Turner Corp., Mountain View, CA, USA). Creatinine was analyzed using the Jaffe rate reaction with a Beckman Synchron CX3 clinical analyzer (Beckman Coulter, Fullerton, CA, USA). Hemoglobin A1C was analyzed via a Tosoh A1C 2.2 plus Glycohemoglobin Analyzer or a Tosoh G7 Automated HPLC Analyzer (Tosoh Medics, Inc., San Francisco, CA, USA). CRP was analyzed via latex-enhanced nephelometry on a Behring Nephelometer Analyzer System (Behring Diagnostics, Inc., San Jose, CA, USA).

Fasting total serum cholesterol and triglycerides were analyzed enzymatically via a Roche/Hitachi Modular P Chemistry Analyzer (Roche Diagnostics Corp, Indianapolis, IN, USA). HDL cholesterol was measured via an adaptation of the customary multistep precipitation reaction.

Biochemistry biomarkers of interest were measured using a Beckman Synchron LX20 and Beckman Coulter UniCel ^®^ DxC800 (Beckman Coulter, Fullerton, CA, USA).

Stata SE/16.0 (StataCorp, College Station, TX, USA) was used for data analysis, as this enabled for the adjustment needed to account for the complex design.

### 2.3. Data Analysis

The data in this cross-sectional study were analyzed for various degrees of lead exposure based on the distribution of BLL within the database. The study examined the role of stress, as measured by AL, on markers of liver injury among individuals differentially exposed to low lead levels.

The geometric mean values of the markers and variables of interest were firstly explored at various quartiles (Q1, Q2, Q3, and Q4) of lead exposure based on the distribution of BLLs in the database. Q1 was defined as those with BLL less than or equal to 0.8 μg/dL and had *n* = 3739 people. Q2 was defined as those with BLL of 0.8–1.21 μg/dL and consisted of *n* = 3765 people. Q3 included those with BLL of 1.21–1.92 μg/dL and consisted of *n* = 3754 people, with Q4 consisting of values 1.92 μg/dL and above and consisting of *n* = 3761 people.

Simple linear regression was performed to examine associations between AL and the markers of interest (AST, ALT, ALP, and GGT) among those exposed to different quartiles of lead. The data was adjusted for age, sex, alcohol consumption, and smoking, as these variables have been shown to alter liver function [34,35,36]. Each exposure–outcome combination was examined in an individual model.

In this study, AL was the dependent variable, with the clinical markers of interest (AST, ALT, ALP, and GGT) being the independent variables. Stata SE 16.0 used for the data analysis which factored in the complex design of the database to ensure the analysis was representative [37] of the US general adult population. The Shapiro–Wilk revealed the necessity of natural log-transformation of variables of interest due to the lack of a normal distribution. P-values less than or equal to 0.05 were considered significant.

## 3. Results

### 3.1. Study Variables among Individuals Exposed to Quartiles of Lead Exposure

The geometric mean values of individuals exposed to lead levels within the four quartiles were analyzed for all critical variables in this study. Results indicated that for most values as lead levels increased, so did the markers of interest. The results can be found in Table 1 below.

### 3.2. Association of AL with Markers of Interest in Low Lead-Exposed Participants

The relationship between AL and the markers among those exposed to quartiles of lead levels was explored using linear regression models. Results indicated that even after adjusting for critical confounding factors, chronic physiological stress significantly altered the liver function among lead-exposed individuals. The results are found in Table 2.

## 4. Discussion

### 4.1. Stress and Liver Health among Those Differentially Exposed to Lead

Exposure to distress can come from several sources such as occupation, family life, emotional problems, and can alter the health of various populations [38,39,40]. When combined with the exposure to lead via water, air, soil, dust, and food [41,42,43], the damage to the health of populations may suffer a combined or synergistic effect [16,26,44,45,46,47].

Allostasis is the process through which individuals maintain physiological balance by altering parameters within the body and matching them to environmental demands [48]. Allostasis, which seeks to adapt to the demands of the environment [49], is similar to homeostasis but is different in that homeostasis defines health as a state in which all physiological parameters must operate within non-changing setpoints (i.e., blood pressure of 120/80 mmHg), with those that cannot be brought down to those values requiring pharmaceutical intervention. With allostasis, individuals can appropriately respond to challenges but, if the challenges are continuous do not turn off, then the body, rather than going to a lower set point, adapts at the higher set point. When the setpoint changes, it is called allostatic load. The ‘wear and tear’ on the body in the adaptation to the new setpoint is critical to understanding the long term health effects [50].

This study sought to examine the effects of chronic stress, as measured by AL, on markers of liver injury among those differentially exposed to lead in the US general adult population. Critical findings within this study indicate that chronic stress, when combined with low-level lead exposure, alters liver markers toward pathology. This study builds on work, which highlights the dangers of low-level lead exposure [51] on human health. It also most critically highlights the risks of chronic unrelenting stress when combined with environmental exposures such as lead and speaks to the dual burden of environmental and social exposures on health.

Men were more represented in higher quartiles of lead exposure when compared to women. This partly due to occupation, where men tend to work in higher lead exposure occupations such as construction and as automobile mechanics [52,53].

The association between AL and ALT and GGT was significant for the three lowest quartiles but was not significant for the highest one. This may potentially be due to several factors such as length of exposure for the lower quartiles when compared to the highest, or may be due to the fact that the highest quartile, though skewed toward lower exposure, had numerous individuals with very high exposure levels.

### 4.2. Limitations

Due to the cross-sectional nature of the study, the exposure and outcome were measured at one point in time and did not allow for examination of temporality. It should be noted that the half-life of lead in blood is roughly one month [54], and it serves as a reflection of acute external exposure to lead. Bone lead levels in concert with blood lead levels would have been useful if they had been available. Another limitation is in the lack of measures of resilience as they may have offered critical insight into the cumulative exposure risk. Finally, the environmental contaminants an individual may be exposed to are multifaceted and may include other toxic metals and organic toxic substances among other exposures. Statistical models may have shown different outcomes if these factors were included. Overall, this study is a critical first step to building larger studies to examine the multifaceted ways in which mixed exposures affect liver health.

## 5. Conclusions

Low-level lead exposure in combination with chronic physiological stress alters hepatic function with increasing levels of lead exposure resulting in worse outcomes. To improve the health of populations, a holistic approach is needed to both decrease their exposure to toxicants and promote behaviors and environments which reduce or prevent chronic stress.

## Figures and Tables

**Table 1:** The geometric mean values of Variables of Interest within Quartiles of Lead Exposure.

Variable	Quartile 1 *	Quartile 2 *	Quartile 3 *	Quartile 4 *	*p*-Value
BLL μg/dL (SE)	0.584 (0.003)	0.992 (0.003)	1.52 (0.006)	3.23 (0.054)	*p* < 0.0001 for all
Age Years (SE)	27.1 (0.337)	35.6 (0.335)	43.5 (0.545)	50.6 (0.599)	*p* < 0.0001 for all
Gender (percent)	Male: 33.9 Female: 66.1	Male: 46.8 Female: 53.2	Male: 55.3 Female: 44.7	Male: 62.4 Female: 37.5	*p* < 0.0001 for all
BMI kg/m2 (SE)	25.9 (0.159)	25.3 (0.204)	26.0 (0.218)	25.9 (0.184)	*p* > 0.05 for all
Allostatic Load (SE)	1.93 (0.058)	2.29 (0.040)	2.59 (0.038)	2.76 (0.046)	*p* < 0.01 for all
AST U/L (SE)	24.5 (0.343)	25.5 (0.203)	26.3 (0.250)	27.2 (0.358)	*p* < 0.05 for all
ALT U/L (SE)	22.9 (0.381)	25.7 (0.407)	26.0 (0.325)	26.2 (0.444)	*p* < 0.0001 for Q1 to Q2, Q3,and Q4
GGT U/L (SE)	20.4 (0.555)	25.2 (0.669)	28.5 (0.537)	33.4 0.829)	*p* < 0.001 for all
Alkaline Phosphatase U/L (SE)	76.9 (1.35)	76.2 (0.775)	73.1 (0.695)	74.7 (0.695)	*p* < 0.05 for Q3 to Q2 and Q1

* The median blood lead level (BLL) for the quartiles were quartile 1—0.60 μg/dL, quartile 2—0.99 μg/dL, quartile 3—1.50 μg/dL, and quartile 4—2.68 μg/dL.

**Table 2:** Simple linear regression—relationship of allostatic load AL with hepatic-markers in Quartiles of lead exposure.

Variable	Quartile 1 *Coef.lnAL Adjusted* (SE) *	*p*-Value	Quartile 2 Coef.lnAL Adjusted (SE) *	*p*-Value	Quartile 3 Coef.lnAL Adjusted (SE) *	*p*-Value	Quartile 4 Coef.lnAL Adjusted (SE) *	*p*-Value
AST U/L	0.178 (0.100)	0.087	0.121 (0.074)	0.111	0.173 (0.051)	0.002	−0.087 (0.044)	0.056
ALT U/L	0.195 (0.073)	0.012	0.165 (0.063)	0.014	0.236 (0.037)	0.0001	−0.007 (0.035)	0.847
GGT U/L	0.149 (0.056)	0.012	0.151 (0.035)	0.0001	0.166 (0.035)	0.0001	0.049 (0.028)	0.094
Alkaline Phosphatase U/L	0.301 (0.102)	0.006	0.167(0.069)	0.021	0.236 (0.057)	0.0001	0.137 (0.061)	0.033

* adjusted for age, sex, smoking and alcohol consumption.
